# Transformer-based advertisement image layout generation and object fidelity optimization

**DOI:** 10.1371/journal.pone.0352418

**Published:** 2026-06-25

**Authors:** Dandan Xu, Kun Hu, Linlu Cai

**Affiliations:** 1 College of Plastic Arts, Daegu University, Gyeongsan-si, Gyeongsangbuk-do, Republic of Korea; 2 College of Computer Science and Mathematics, Fujian University of Technology, Fuzhou, Fujian, China; 3 Hanyang University ERICA Campus, Ansan-si, Gyeonggi-do, South Korea; 4 Xing’an Polytechnic University, Dulin West Street, Horqin Right Front Banner, Xing’an League, Inner Mongolia, P.R. China; University of Engineering and Technology Taxila Pakistan, PAKISTAN

## Abstract

The layout of the image and the positional distribution of the objects directly affect the audience’s visual focus and the effect of the message conveyed. By setting up a specific layout structure, the viewer’s attention can be more focused on the product launched or the moral of the advertisement. Existing work related to position-controllable text-to-image generation has made great progress in generating results on simple images. However, when generating images of complex scenes, the quality is often poor. This can result in the model failing to accurately convey the message of the advertisement when used to generate advertisements. To address these limitations, we propose LAYOBJ-GAN, a novel two-stage framework for layout-controllable advertisement image synthesis. Unlike prior work, our method explicitly models background layouts jointly with object layouts during the text-to-layout generation stage, enabling comprehensive spatial planning of complex scenes. Technically, we introduce a Transformer-based sequence-to-sequence layout generator that learns long-range dependencies between textual descriptions and both background and object regions, which has not been explored in previous advertisement-oriented text-to-image frameworks. In the layout-to-image stage, we further propose a fine-grained text–layout interaction normalization module (TL-Norm) that enables object knowledge transfer from a pre-trained category-to-image model, allowing object appearance to be adaptively modulated by textual context and layout constraints. Extensive experiments on MS-COCO and a high-definition advertisement dataset (AsHQ-10K) demonstrate that LAYOBJ-GAN significantly outperforms seven state-of-the-art methods in image quality, layout controllability, and semantic object accuracy. These results confirm the effectiveness of explicitly modeling background layouts and transferring object-level generative knowledge for complex advertisement image synthesis.

## Introduction

In computer vision and cross-modal analysis, text generation of images is a crucial problem [1]. Using descriptions in plain language, the goal seeks to produce realistic and semantically consistent images. The text-to-image (T2I) task offers a flexible and natural interface for conditional image generation by letting users explain visual notions using natural language. For AI-generated ads, text-to-image (T2I) technology is a crucial building block. By inputting natural language descriptions, AI can automatically generate visual content that meets the needs, such as product demonstrations, scene building, or branding graphics [2]. This technology greatly improves the efficiency of ad creation and reduces labor costs. It also allows advertisers to quickly iterate creative solutions. For example, advertisers only need to input “a cool drink on a sunny beach with palm trees and blue sky in the background,” and the AI-generated advertisement can generate high-quality images to meet the brand’s tone and marketing objectives.

With the development of deep learning [3,4], the core strengths of text-to-image generation technology are precise content control, efficient creative generation, and personalization. By adjusting the text description, T2I can precisely control the objects, style, and atmosphere in the advertisement. Without the intervention of professional designers, T2I can quickly generate a wide range of visual solutions to choose from. At the same time, T2I is able to generate differentiated ad content for different audience groups to enhance the effectiveness of the campaign.

The controllability of the middle position of text-generated images has become a research focus in recent years. This is simultaneously reflected in the background layout of AI-generated advertisements. Background layout is a key factor affecting the quality and communication effect of advertisements [5]. At the visual level, a reasonable layout can guide the audience’s attention and highlight the core information (e.g., products, promotional texts). Ensure that the subject matter of the advertisement is clear and understandable through the arrangement of objects and text. A balanced composition (e.g., thirds, symmetrical layout) enhances the professionalism and appeal of the advertisement in terms of aesthetic appeal. In addition, the background generally accounts for more than 70% of the image, which directly affects the realism and immersion of the advertisement. For example, an e-commerce advertisement will look monotonous or unrealistic if it lacks background details (e.g., home environment). Fixed-style backgrounds (e.g., minimalist, retro) can also strengthen brand visual identity.

In recent years, Generative Adversarial Network (GAN)-based approaches have shown encouraging results in location-controlled text-generated image tasks [6]. Since user-specified layouts are often not comprehensive enough and it is sometimes desirable to provide systems that can provide predefined layouts, the location-controlled text-to-image generation task is generally solved by a two-stage scheme [7]. Text-to-layout generation is the first step, while layout-to-image generation is the second. According to this study, the effectiveness of current approaches is mostly restricted to simple datasets like flowers and birds, and creating intricate, real-world images like MS-COCO is still very difficult [8,9]. First, current methods tend to model object-centric layouts, and the two-stage approach, despite being able to generate more realistic object-centric images than end-to-end text-generated image generation methods, is able to generate. These approaches, however, continue to ignore background-related layouts, which make up 70% of the regions in images and 38.2% of the nouns in text descriptions [10]. A substantial amount of background information in the written description is lost by current approaches that solely rely on object arrangement. In order to enhance the overall quality of the generated photographs, the background can be regarded as a component of the foreground object-based layout modeling. This is especially crucial for activities involving AI-generated advertisements. Beyond the specific domain of text-to-image and layout-based generative models, recent advances in deep learning have explored attention mechanisms, hybrid architectures, and transfer learning strategies across diverse image analysis tasks. For example, hybrid deep learning frameworks that combine attention and statistical validation have demonstrated strong performance in medical image segmentation tasks, highlighting the value of attention mechanisms for capturing complex structures and feature interactions. In image classification and pathological analysis, comprehensive studies of deep models for diabetic retinopathy detection show how model design and data handling can critically affect robustness across conditions and classes. Methods involving adaptive kernel attention and feature selection have also been investigated in the context of neurodevelopmental disorder classification, illustrating the cross-domain relevance of feature weighting and model explainability in deep architectures. These works collectively underscore the importance of architectural enhancements, attention mechanisms, and evaluation rigor, which inform the design choices in controllable generative frameworks like ours, even when the application domain differs.

Knowledge learning of various objects using controlled text generation picture models is hampered by a lack of linked image-text data. Nevertheless, there is a substantial cost associated with directly gathering additional paired image-text data with region annotations. In order to reduce object distortion in generated images, it seems sense to investigate alternative image production models as a possible source of object information. Current category-to-image generation models based on large-scale prediction can generate realistic images for a given object category [11], such as 1000 categories in ImageNet. Another way to look at the category-to-image creation process is as a condensed form of the layout-to-image generating process, in which the resulting target picture only contains one object. To make it easier to generate each object in complex images, this work investigates the transfer of knowledge from a predicted single-object image generation model to text-generated images.

Overall, in order to solve the above problems of unrealistic backgrounds and distorted objects in AI-generated advertisements, a new two-stage position-controllable text-generated image method is proposed in this study. The method is called Background and Object Generation Adversarial Network (LAYOBJ-GAN) to comprehensively improve the generation quality and position controllability of background and promotional item regions. Two new sub-models are designed: 1) in the background layout generator, the text-to-layout phase is redesigned with a Transformer-based architecture that predicts a comprehensive layout that combines backgrounds with objects; and 2) the layout-to-image phase is generated more easily in the picture generating session thanks to an object knowledge transfer module that uses object knowledge from a single-object image production model. Experiments have shown that LAYOBJ-GAN, designed by combining these two components, is capable of generating complex and diverse real-world images. Unlike prior two-stage frameworks that primarily focus on object-centric layout prediction, the proposed method introduces a unified formulation that jointly models background regions and object instances as a coherent layout sequence. This enables full-scene spatial planning rather than partial object placement. Furthermore, while conditional normalization and attention mechanisms have been explored in prior works, the proposed TL-Norm differs in that it explicitly integrates text-attentive category embeddings into instance-level normalization, allowing object appearance to be dynamically modulated by both layout constraints and contextual semantics. This design bridges category-to-image priors with layout-conditioned generation, which has not been systematically explored in previous layout-controllable text-to-image frameworks. The research made in this paper is as follows.

(1) A location-controlled image generation method for text is proposed: background and object generation adversarial network (LAYOBJ-GAN). The method effectively improves the generation quality of existing methods in the background and promotional item regions.
(2) This study created a Transformer-based sequence-to-sequence model to create layouts with backgrounds, which directs the creation of text-to-complex layouts, thereby easing the challenge of creating advertisement images for intricate scenes.
(3) By transferring knowledge from other image generation tasks, the generation quality of objects under limited paired image-text data can be effectively improved. The suggested approach achieves the optimal image-text alignment performance and significantly beats prior methods in terms of image quality measures on a self-built dataset of AI-generated marketing graphics.

## Related work

In the advertising industry, the dynamic evolution of advertising strategies has accelerated the development of AI, fundamentally altering the interaction patterns between brands and their target audiences. Advertisers widely utilize AI technology to create personalized user experiences, achieve precise audience targeting, and accelerate decision-making processes [12].

The deep integration of AI and advertising has become a key indicator of the paradigm shift in modern marketing strategies. Experts believe that introducing AI technology into the advertising field can reshape many parts of the advertising process, including advertising research, planning, creative conception, copywriting, media arrangement and procurement, and effectiveness evaluation. Furthermore, AI technology has room for application in areas such as direct mail advertising copywriting, precise advertising delivery, accurate target audience positioning, and future trend prediction [13]. Currently, this technology is comprehensively revolutionizing the interaction model between brands and audiences, ushering in a new chapter in advertising communication.

### CNN and GAN-based generation methods

CNN-based architectures have played a foundational role in image understanding and generation tasks. For example, GoogLeNet [14] introduces inception modules that enable multi-scale feature extraction, significantly improving performance in complex visual recognition tasks. In addition to conventional CNNs, Capsule Networks (CapsNet) [15] have been proposed to better model spatial hierarchies and part–whole relationships. Although these methods demonstrate strong capabilities in feature extraction and representation learning, they primarily focus on classification or detection tasks. In contrast, our work addresses layout-controllable image generation, where both spatial planning and semantic alignment must be jointly modeled. This highlights the necessity of integrating Transformer-based sequence modeling with layout-aware generation mechanisms.

Combining recurrent neural networks (RNNs) with GANs to generate videos based on text is a method that combines sequence modeling with adversarial learning [16]. The generator RDN of RD-GAN, combined with a 3D-CNN discriminator, can generate realistic videos based on text semantics [17]. The MoCoGAN framework decomposes random vectors into fixed content and motion parts, and uses image and video discriminators to achieve unsupervised motion and content decomposition, enabling the generation of videos with different motion or content combinations [18].

The CogVideoX model can leverage technologies such as 3D full attention mechanism to enhance semantic alignment and temporal coherence [19]. There are also studies using conditional GAN, semantic – aware recurrent GAN, etc., which optimize the temporal coherence and semantic matching effects of video generation through specific network structures and loss functions [20].

Beyond conventional CNN architectures, researchers have explored attention-enhanced and Capsule Network-based frameworks to improve feature representation and contextual modeling. Capsule Networks preserve hierarchical relationships between features and have demonstrated strong performance in complex pattern recognition tasks. For example, Boruah and Das proposed CaDenseNet, which integrates capsule networks with attention mechanisms [21–24].

### Transformer-based generation methods

The Transformer-based text-to-video generation approach represents a frontier in the field of multimodal artificial intelligence [25]. Its core lies in harnessing the powerful sequence modeling capabilities of Transformers to establish a mapping from textual semantics to dynamic visual content. In the research of text-to-video generation based on Transformer, numerous models have achieved technological breakthroughs from different perspectives. CogVideo is the first open-source general large-scale pre-trained model. Based on a 9B-parameter Transformer [26]. It addresses the issues of high computational costs and scarce datasets through inheriting CogView2 and adopting a multi-frame rate hierarchical training strategy, demonstrating excellent performance in evaluations. EasyAnimate extends the DiT framework to 3D video generation, introducing a hybrid motion module and Slice VAE technology [27]. This not only ensures frame-to-frame coherence but also reduces memory usage, establishing a complete video production ecosystem. Meta’s Movie Gen employs a pure Transformer architecture [28]. Through flow matching and multi-stage training, it outperforms models like Sora in text-to-video and text-to-image generation.

Moreover, Google has also made significant achievements in this field. Make-A-Video can directly generate videos by combining diffusion models and Transformer temporal modeling without the need for text-to-image fine-tuning [29]. Phenaki utilizes ultra-long sequence Transformers and spatio-temporal attention mechanisms to enable the generation of videos with thousands of frames, excelling in handling complex scene transitions [30]. Imagen Video leverages the T5 text encoder and video diffusion Transformer, generating videos with high color fidelity through a 3D diffusion process [31].

In addition, other innovative models continue to emerge. ByteDance’s T2V-GPT combines a GPT-like autoregressive Transformer architecture with 3D convolutions, generating videos frame by frame in sequence and demonstrating outstanding long-term temporal processing capabilities. VideoGPT combines VQ-VAE and Transformer, verifying the superiority of autoregressive Transformers over GANs in text-to-video generation and providing important insights for early research [32].

### Diffusion model-based generation methods

Video generation methods based on diffusion models achieve controllable generation of high-resolution, long-term coherent videos from conditional inputs like text or images by extending image diffusion models to spatiotemporal dimensions, integrating multi-stage workflows, and training with large-scale video-text datasets [33]. In the field of text-to-video generation, early diffusion models like VDM (Video Diffusion Model) pioneered the extension of 2D image diffusion architectures to 3D U-Net frameworks [34–36]. By jointly training on image and video datasets, VDM enables fundamental video generation—for instance, converting text prompts like “a premium coffee machine brewing a smooth latte” into coherent product demonstration videos. Make-A-Video reduces reliance on video-text paired datasets by inventing movement patterns from unsupervised video information and learning visual-semantic associations from image-text pairs [37]. This allows efficient generation of advertising variants, such as adapting to marketing phrases like “summer beverages + beach scenes + refreshing taste” for FMCG brands.

Hierarchical diffusion architectures have become pivotal for optimizing generation quality and efficiency. Video LDM employs a three-stage pipeline: keyframe generation using temporal attention and 3D convolutions, frame rate enhancement via mask sampling-based interpolation, and resolution upscaling to 720P+ through super-resolution modules [38]. This workflow compressed the production cycle of Coca-Cola’s fully AI-generated advertisement from weeks to 3 days. Conversely, Imagen Video constructs a seven-stage cascaded diffusion model that consists of one base video generator and three groups of spatial-temporal super-resolution modules. It achieves 4K-level outputs using techniques such as classifier-free guidance, thereby meeting the high-definition requirements for automotive TV commercials [39].

For dynamic scene modeling, models like Sora introduce Diffusion Transformer architectures, resolving complex motion coherence issues through spatiotemporal block encoding and dynamic attention mechanisms [40,41]. This enables precise simulation of physical movements, such as the pouring trajectory of liquid cosmetics or the operation of mechanical products in advertisements. VidRD proposes a “Reuse and Diffuse” framework, generating continuous frames by iteratively reusing latent representations to create videos that exceed 30 seconds in length, making it ideal for brand story-telling videos [42]. GridDiff reimagines videos as grid images, using 2D U-Nets for spatiotemporal processing to significantly reduce computational costs. This enables real-time generation of e-commerce live-stream ads, such as clothing try-on combined with promotional slogans, with the cost of generating a single video kept under control [43].

## Method

**Fig 1:**
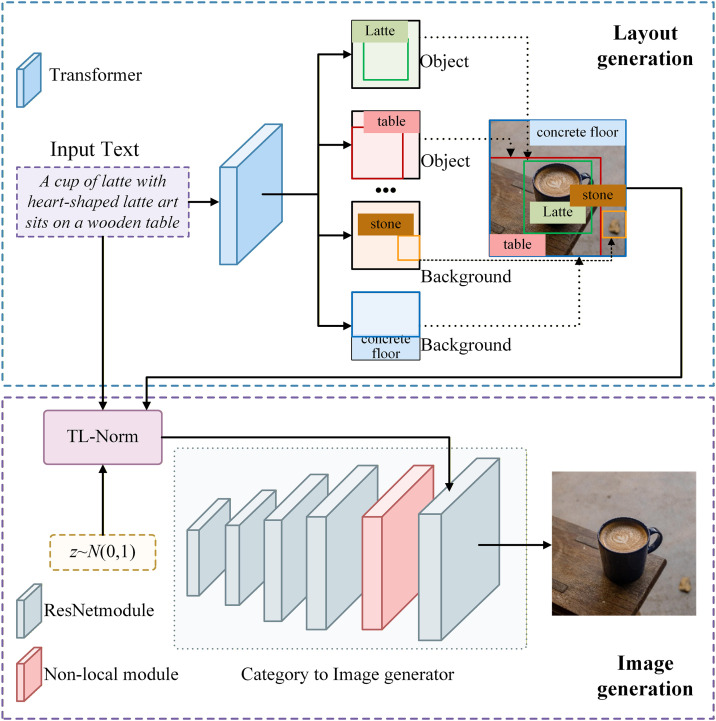
The overall process of LayObj-GAN.

The two steps of our suggested method are layout generation and image generation, as seen in Fig 1. First, a Transformer-based seq2seq model is developed to produce backdrop-aware layouts by offering precise and detailed spatial semantic recommendations for background generation. Then, in order to alleviate the object distortion problem, a fine-grained text-layout interaction module, TL-Norm, is introduced.

### Transformer-based sequence-to-sequence background layout generation

**Fig 2:**
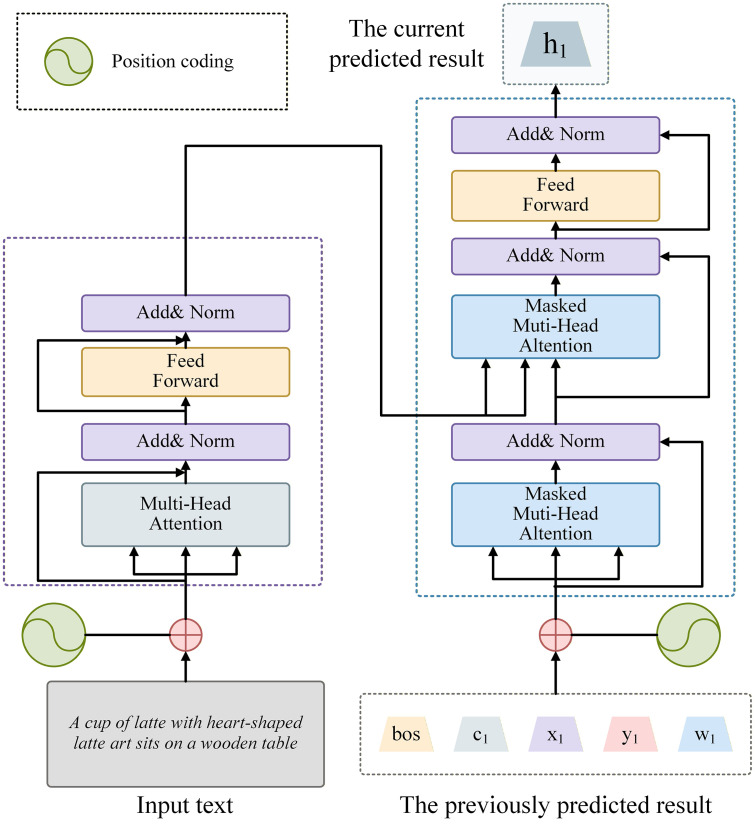
Transformer-based sequence-to-sequence background layout generation.

Since a layout may be defined as a discrete sequence, text-to-layout generation can be viewed as a sequence-to-sequence generation process. The transformation process from text to object and backdrop layouts is then learned using a Transformer-based model, as seen in Fig 2.

Text Representation. Since embeddings derived from images may include irrelevant details, such as color information, which are unnecessary for layout generation, this paper avoids using text encoders pre-trained on image-text matching tasks for layout generation. Instead, a Transformer encoder with initialization parameters is used for text representation. This learning is able to learn better layout-relevant text representations and exclude the interference of layout-irrelevant information. The representation of the text is optimized end-to-end based on the loss generated by the layout.

Text-to-layout generator. To model a discrete sequence of layouts conditioned on textual inputs, this paper employs a Transformer-based encoder-decoder architecture. The Transformer design excels at long-range dependency modeling and inference. The encoder consists of multiple parallel self-attention modules, each with a multi-head self-attention layer and a feed-forward completely linked layer. Both layers follow residual connectivity and layer normalization, thus ensuring stability of model training while effectively fusing information between layers. In the decoder part, which also consists of several modules with the same structure, it is responsible for generating the layout sequence from the coded information step by step. The decoder module receives the information output from the encoder and, in contrast to the encoder structure, adds a multi-head cross-attention layer in addition to the feedforward and multi-head self-attention layers. This cross-attention mechanism can effectively integrate the correlation between the semantic information of the textual input and the layout generation sequence, ensuring that the decoder can generate layout content that is highly consistent with the textual conditions.

Layout Representation. This work uses a similar approach to the one in Layout-Transformer for layout sequence representation. A layout (L) consists of several (N) bounding boxes (B), each represented by its category label (c), upper-left corner position (x,y), width and height (w,h) as shown in Equation 1.


$$\begin{array}{c} L=\left[B_{\text{1}},B_{\text{2}},\ldots ,B_{N}\right],B_{i}=\left[c_{i},x_{i},y_{i},w_{i},h_{i}\right] \end{array}$$ (1)

The order of the bounding box is determined by y. In addition, the successive positions (x,y,w,h) are discretized as integers from 0 to 127, appended with two tokens $s_{bos}$, $s_{eos}$ indicating the start and end of the layout. Equation 2 illustrates how the layout is expressed as a continuous sequence up until this point.


$$\begin{array}{c} L=\left[s_{bos},c_{\text{1}},x_{\text{1}},y_{\text{1}},w_{\text{1}},h_{\text{1}},\ldots ,c_{N},x_{N},y_{N},w_{N},h_{N,}s_{eos}\right] \end{array}$$ (2)

Inference. In the inference phase, the labeling generation process for discrete sequences is autoregressive, i.e., the structure generated at each step depends on the input conditional text and previously generated layout information and user-specified layout information. Specifically, autoregressive generation first inputs the conditional text into the model to obtain contextual information related to the layout content. The model then generates markers for the layout sequence step-by-step based on this contextual information. In each step, the model takes as input the previously generated layout markers, thus ensuring that the generated layout sequence maintains continuity. If the model generates the last box incomplete, then the last incomplete box is ignored, and only the layout information generated from the complete box is used for the second stage of image generation.

### Image generation based on object knowledge migration with fine-grained text-layout interaction

Creating realistic images from textual descriptions and background-aware layouts is the main goal of the layout-to-image phase. In order to do this, this study suggests using TL-Norm to adaptively transfer the layout-to-image phase’s object BigGAN information from the ImageNet pre-trained category-to-image model. This trained model can produce high-quality photos with 1,000 different item classifications.

Image generation based on object knowledge migration and fine-grained text-layout interaction is divided into two main steps. First, for the ability to migrate categories to generate image models in the layout generation image task, and second, for the fine-grained text-layout interaction module TL-Norm.

When only one bounding box exists in the layout and that bounding box covers the entire image, the layout-to-image generation process can be reduced to a category-to-image generation task. Based on this observation, we hypothesize that a well-trained category-to-image model can effectively assist layout-to-image generation, especially in the object generation session. We then describe how the conditional batch normalization (CBN) layer can be modified to fit the category-to-image model to the layout-to-image generation task.

CBN is a frequently used layer for conditioned control in conditioned picture generation. Equation 3 takes an input feature map $x\in R^{N\times C\times H\times W}$ and first normalizes each channel to a mean of zero and unit standard deviation.


$$\begin{array}{c} {\overset{―}{x}}_{nchw}=\frac{x_{nchw}-\mu_{c}(x)}{\sigma_{c}(x)} \end{array}$$ (3)

$$\begin{array}{c} \mu_{c}(x)=\frac{\text{1}}{NHW}\sum_{n,h,w}x_{nchw} \end{array}$$ (4)

$$\begin{array}{c} \sigma_{c}(x)=\sqrt{\frac{\text{1}}{NHW}{\left(x_{nchw}-\mu_{c}(x)\right)}^{\text{2}}} \end{array}$$ (5)

Equation 6 illustrates how conditional information cond is then utilized to anticipate the scale and bias in order to adjust the normalized features.


$$\begin{array}{c} {\dot{x}}_{nchw}=\left(\lambda (cond)+\text{1}\right){\overset{―}{x}}_{nchw}+\beta (cond) \end{array}$$ (6)

where the function λ and the function β are realized through MLP or convolutional layers. The conditional information can thus be used by the CBN to effectively regulate the generation process.

The CBN module CBN for Layout-to-Image: Instance-Specific and ISLA-Norm, which has been recommended in the literature [44], enables fine-grained layout control for layout-to-image production. It consists of the following four steps: label embedding, instance-specific projection, mask Prediction and computation of ISLA λ(L) andβ(L).

First, embedding is processed for each instance category in the layout, represented by an embedding vector $\overset{\to }{d}$. This vector combines the embedding representation $d^{e}$ of the instance category and the instance-specific style noise ${\overset{\to }{d}}^{noise}$ to represent both the category information and the random style changes.

Labeled embeddings are used in CBN to compute the instance-specific scale λ(b) and deviation β(b) for each bounding box. Specifically, for each bounding box b, the labeled embedding vector $\overset{\to }{d}$ is mapped to the scale λ(b) and deviation β(b) through the CBN layer. Thus, the feature distribution of each instance can be adjusted in a targeted manner during feature normalization. This step allows the model to perform dynamic feature control based on instance characteristics.

Separation of foreground and background regions for each bounding box is performed using a mask predictor. The mask predictor consists of a series of convolutional layers, an upsampling layer, and an instance normalization module. A soft mask $M_{b}$ is output through a sigmoid activation function to provide a probability distribution of the foreground region for each bounding box. The soft mask $M_{b}$ can help the model to focus on the foreground objects within the bounding box during feature adjustment.

The λ(b) and β(b) of each instance are extended to the bounding box region and multiplied with the corresponding soft mask $M_{b}$ to limit the scope of action to the foreground region. Next, the results of these modulations within the bounding box are combined into feature control parameters λ(L) and β(L) for the entire layout by summing over all instances and applied to the full map generation process. In this way, ISLA−Norm is able to realize the fine control of different instances and layout regions so that the generated image fits the structural information of the input layout more closely.

CBN for category-to-image: As illustrated in Fig 1, the category-to-image model is composed of multiple ResBlocks and a nonlocal block. Each ResBlock includes several convolutional layers to capture the nonlinear transformation across stages. The conditional input comprises the category embedding and a low-dimensional noise vector. The normalized features are modulated using predicted scale ($\lambda (cls)\in R^{N\times C}$) and bias ($\beta (\text{cls})\in R^{N\times C}$) parameters.

The category-to-image model is used for layout to images. After the broadcast operation, the scale and bias are represented as $\lambda (cls)\in R^{N\times C\times H\times W}$ and $\beta (cls)\in R^{N\times C\times H\times W}$, where each position has the same features. Thus, λ(cls) and β(cls) can be replaced with λ(L) and β(L) computed by ISLA−Norm in the category-to-image model to facilitate layout-to-image generation.

This is followed by the fine-grained text-local interaction module TL-Norm. In order to achieve optimal layout-image consistency, this study also takes into account the textual context during layout-to-image generation. The scales λ and β, which are calculated from sentence embeddings and layouts, can be added together to directly integrate textual information. The representation is shown in Equation 5.


$$\begin{array}{c} \overset{sum}{\underset{L+T}{\lambda }}=\lambda (L)+\lambda \left(sent_{emb}\right), \end{array}$$

$$\begin{array}{c} \overset{sum}{\underset{L+T}{\beta }}=\beta (L)+\beta \left(sent_{emb}\right) \end{array}$$ (5)

If noise is ignored in this structure, the previous mask $M_{b}$ and the instance-specific projections λ(b) and β(b) that are calculated from the same instance category are the same in various texts. However, in practice, the same instance categories often present different meanings and appearances in different textual contexts. For example, suppose the input sentences are “a girl reading” and “a girl walking down the street with a balloon.” In the first description, the “girl” is more focused on her static behavior, and the resulting image might show her sitting quietly reading. While the latter sentence emphasizes her interaction with the object, the image might highlight the balloon in her hand and her posture on the street. Therefore, in order to achieve accurate generation, the instance category information should be more closely related to the textual context during the generation process so as to adjust the gestures, shapes, and expressions of the instances according to the context.

TL-Norm: To do this, TL-Norm is suggested in this paper. Category-text attention calculates the instance’s contextual text information. After encoding with a pre-trained text encoder, two types of word embeddings can be obtained, assuming that the input text contains T word tokens. They are contextual word representations from the text encoder’s final layer ($W^{c}=\left\{\overset{c}{\underset{\text{1}}{w}},\overset{c}{\underset{\text{2}}{w}},\ldots ,\overset{c}{\underset{T}{w}}\right\}\in R^{T\times D}$) and context-independent word embeddings from the initial input embedding layer ($W^{e}=\left\{\overset{e}{\underset{\text{1}}{w}},\overset{e}{\underset{\text{2}}{w}},\ldots ,\overset{e}{\underset{T}{w}}\right\}\in R^{T\times H}$), respectively. The input embedding layer $C=\left\{c_{\text{1}},c_{\text{2}},\ldots ,c_{N}\right\}\in R^{N\times H}$ can additionally represent the layout’s backdrop and object category embeddings. The instance category embeddings for text attention can be represented by the following equation, which first calculates the attention score between the category embeddings and the context-independent word embeddings, as shown in Equation 6.


$$\begin{array}{c} s_{i,j}=\frac{exp\left({\left(c_{i}\right)}^{T}\overset{e}{\underset{j}{w}}\right)}{\sum_{k=\text{1}}^{T}exp\left({\left(c_{i}\right)}^{T}\overset{e}{\underset{j}{w}}\right)} \end{array}$$ (6)

This is then expressed in terms of attention score-weighted context words, as shown in Equation 7.


$$\begin{array}{c} h_{i}=\sum_{k=\text{1}}^{T}s_{i,j}\overset{c}{\underset{k}{w}} \end{array}$$ (7)

Finally, replace $\overset{e}{\underset{i}{d}}$ with $\overset{e}{\underset{i}{d}}+h_{i}$in the original ISLA-Norm module. The original ISLA-Norm’s other operations are identical. Therefore, the specific four steps of TL-Norm are as follows. First, each instance category in the layout is represented by a vector d, which combines the text-attentive category embedding, namely $d^{e}+h_{i}$ and the style noise $d^{noise}$, where $d^{e}$ is the category embedding. The foreground and background inside the bounding box are then separated using a mask predictor. Several convolutional and upsampling layers make up this predictor, and the final layer is a sigmoid function that produces a soft mask ${\text{M}}_{\text{b}}$ for every bounding box. Then, using the identified embeddings, the conditional batch normalization computes the instance-specific scale λ(b) and deviation β(b) of each bounding box. Finally, λ(b) and β(b) of each instance are expanded and multiplied with the soft mask for the corresponding bounding box, and these are then combined through summarization to produce the layout-aware features with textual attention.

**Fig 3:**
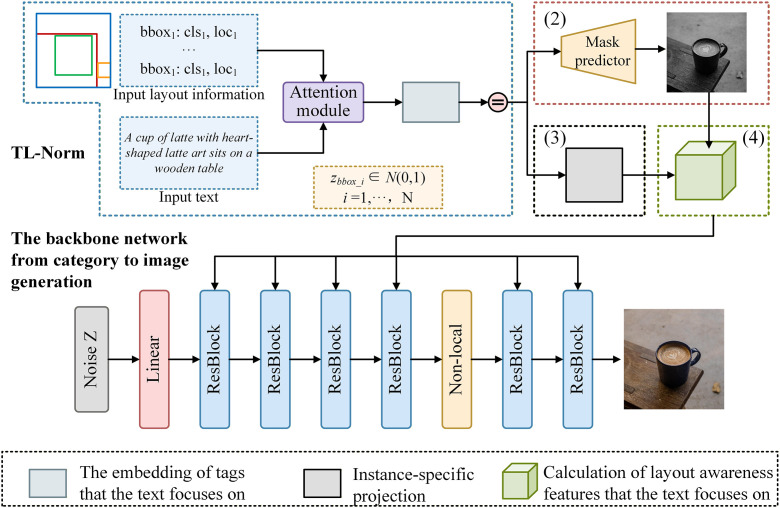
The generator structure of LayObj-GAN.

**Fig 4:**
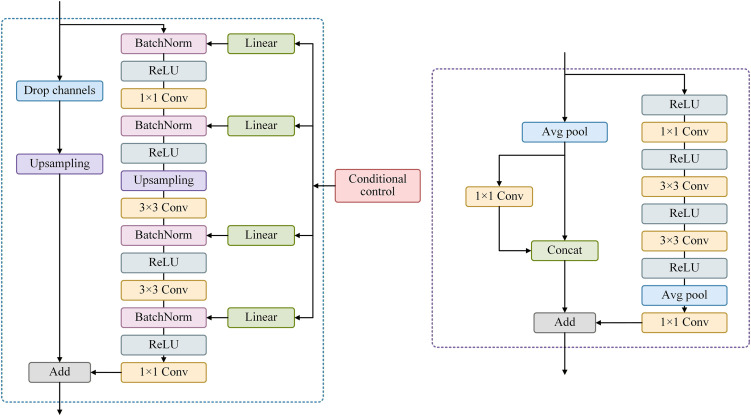
Specific composition of ResBlock in LayObj-GAN’s generator and discriminator.

Specialized architecture for generators and discriminators. The generator’s intricate construction is depicted in Fig 3. The category-to-image generator backbone network and the TL-Norm are the two primary components of the generator. In order to guarantee that the generative power of the original network is maximized and that the local details of the generated image are globally consistent, the TL-Norm module is utilized to modify the modulation parameters of the spatial features during the generation process. The image generator backbone network is derived from a pre-trained model. Through a series of convolutional and anti-convolutional layers, the spatial structure and content details of the image are gradually constructed, and the perception of global information is realized through the non-local network structure. The specific structure of ResBlock in the generator and discriminator is depicted in Fig 4.

**Fig 5:**
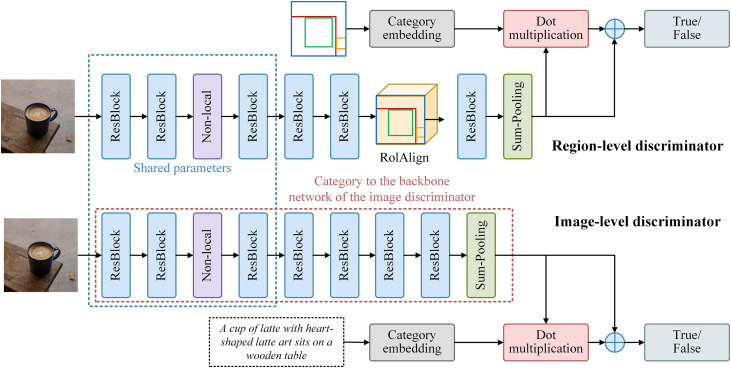
Discriminator structure of the LayObj-GAN.

Fig 5 shows the detailed architecture of the discriminator. In this paper, we use a region-level discriminator and an image-level discriminator to distinguish between object- and image-level features, respectively. The two discriminators jointly model the combined local-global information of the image. The backbone network of the image-level discriminator is derived from the pre-trained category-to-image discriminator, which draws on the discriminative representation capabilities of the pre-trained models. The first four blocks are shared by the region-level discriminator and the image-level discriminator, in which the non-local network structure is also introduced to realize more accurate discrimination. In the region-level feature discriminator network, the local area features of the image are obtained by ROI pooling. Then the latent information related to the category is dot product and normalized to get the final discriminative result. The image-level discriminator, on the other hand, focuses on the discrimination of the overall quality of the image and models the authenticity of the correlation between the generated image and the text by performing dot product operations with the text features. This approach ensures the authenticity and consistency of individual objects in the generated image and can effectively detect subtle differences between local features and specified categories. In contrast, image-level discriminators focus mainly on the overall image quality discrimination. This multi-level discriminator, combining local and global, text and image, can enhance the high-quality consistency of the generated images in terms of structure, content, and semantics.

### Model training

The LayObj-GAN model is based on a two-stage model. The basic flow of its training is shown in Algorithm 1, and the loss function for each stage is described below.

Algorithm 1. The Basic Process of LayObj-GAN Training

1. **Input:** (T,L,I), $G_{L}$, $G_{I}$,$D_{I}$

2. **Output:** trained $G_{L}$,$G_{I}$

3. **#** stage 1: train$G_{L}$

4. **while not** converged $G_{L}$ **do**

5.   sample ($t_{j},I_{j},i_{j})$ from(T,L,I)

6.   **update** $G_{L}$ ←${Loss}_{layout}=-\frac{\text{1}}{K}\sum_{i=\text{1}}^{K-\text{1}}KL\left(p_{i}||{\hat{p}}_{i}\right)$

7. **end while**

8. **#** stage 2: train $G_{I}$(with $D_{I}$)

9. **while not** converged $G_{I}$ **do**

10.   sample $\left(t_{j},I_{j},i_{j}\right)$ from(T,L,I)

11.   **update** $G_{I}$ ←$L_{G}=\lambda_{DAMSM}L_{DAMSM}+\lambda_{img}E_{img\sim \overset{g}{\underset{img}{P}}}\left[-D\left(img|s\right)\right]+\lambda_{obj}E_{obj\sim \overset{g}{\underset{obj}{P}}}\left[-D\left(obj|y\right)\right]$

12.   **update** $D_{I}$ ←$L_{D}=\lambda_{obj}\left(E_{obj\sim \overset{g}{\underset{obj}{P}}}max\left[\text{0},\text{1}+D\left(obj|y\right)\right]+E_{obj\sim \overset{r}{\underset{obj}{P}}}max\left[\text{0},\text{1}-D\left(obj|y\right)\right]\right)+\lambda_{img}\left(E_{img\sim \overset{s}{\underset{img}{P}}}max\left[\text{0},\text{1}+D\left(img|s\right)\right]+E_{img\sim \overset{r}{\underset{img}{P}}}max\left[\text{0},\text{1}-D\left(img|s\right)\right]\right)$

13. **end while**

Loss function of layout generator. In this paper, the layout generator is trained using a teacher-forcing technique. To increase convergence and generation accuracy, actual markers are supplied into the model at each stage of the layout marker production process rather than ones that have already been created. To mitigate the overfitting problem on limited datasets, label smoothing loss is introduced to avoid overconfidence of the model on a single category, allowing the generator to have a better generalization ability. This loss computes the KL scatter between the predictive distribution $p_{i}$ and the smoothed ${\hat{p}}_{i}$ of the one-shot distribution, as shown in Equation 8.


$$\begin{array}{c} {Loss}_{layout}=-\frac{\text{1}}{K}\sum_{i=\text{1}}^{K-\text{1}}KL\left(p_{i}||{\hat{p}}_{i}\right) \end{array}$$ (8)

where K denotes the number of categories.

The picture generator’s loss function. The image generator’s objective is to generate high-quality images within each bounding box (position controllability) and actual images that are semantically compatible with the text input. The discriminator and DAMSM, a pre-trained image-text matching model, provide the first portion of the generator loss. Semantic embedding of generated images and text explanations is provided by DAMSM. In this way, the generator may be guided to learn the semantic content of the text input by directly computing the loss of similarity between the image and text in the embedding space. Furthermore, the discriminator assesses the created image’s quality directly from the standpoint of true-false image discrimination. Equation 9 illustrates that the object-level discriminator is responsible for the second portion of the loss.


$$\begin{array}{c} L_{G}=\lambda_{DAMSM}L_{DAMSM}+\lambda_{img}E_{img\sim \overset{g}{\underset{img}{P}}}\left[-D\left(img|s\right)\right]+\lambda_{obj}E_{obj\sim \overset{g}{\underset{obj}{P}}}\left[-D\left(obj|y\right)\right] \end{array}$$ (9)

By differentiating between actual and false images and objects, the discriminator, on the other hand, aims to increase the generated images’ realism. As shown in Equation 10.


$$\begin{array}{cc} L_{D}= & \lambda_{obj}\left(E_{obj\sim \overset{g}{\underset{obj}{P}}}max\left[\text{0},\text{1}+D\left(obj|y\right)\right]+E_{obj\sim \overset{r}{\underset{obj}{P}}}max\left[\text{0},\text{1}-D\left(obj|y\right)\right]\right)\\ & \;\;+\lambda_{img}\left(E_{img\sim \overset{s}{\underset{img}{P}}}max\left[\text{0},\text{1}+D\left(img|s\right)\right]+E_{img\sim \overset{r}{\underset{img}{P}}}max\left[\text{0},\text{1}-D\left(img|s\right)\right]\right)\; \end{array}$$ (10)

where $L_{DAMSM}$, $\lambda_{obj}$, and $\lambda_{img}$ are hyperparameters used to weight the respective losses. $\overset{g}{\underset{obj}{P}}$ and $\overset{r}{\underset{obj}{P}}$ denote the generated and real object distributions, respectively. $\overset{s}{\underset{img}{P}}$ and $\overset{r}{\underset{img}{P}}$ denote the generated and real image distributions, respectively.

The MS-COCO-14 benchmark has an image resolution of 256 by 256 pixels. Nevertheless, the majority of the open-source category-to-image generators and discriminators that are now in use are trained on images that have a resolution of just 128 by 128 pixels. Due to the available hardware resource limitations, it is not possible to train a model that generates 256 by 256 images on ImageNet. Therefore, the proposed layout-to-image model is first trained at a 128 by 128 setting using the pre-trained category-to-image model and then scaled up to 256 by 256 using an asymptotic growth scheme. Specifically, the loss functions of the generator and discriminator combine low- and high-resolution loss weights during the gradual resolution scaling process. The loss functions are shown in Equation 11 and 12.


$$\begin{array}{c} L_{G}=L_{G\text{2}\text{5}\text{6}}+max\left(\text{0},\text{1}-\frac{cur_{step}}{fade_{step}}\right)L_{G\text{1}\text{2}\text{8}} \end{array}$$ (11)

$$\begin{array}{c} L_{D}=L_{D\text{2}\text{5}\text{6}}+max\left(\text{0},\text{1}-\frac{cur_{step}}{fade_{step}}\right)L_{D\text{1}\text{2}\text{8}} \end{array}$$ (12)

where $cur_{step}$ denotes the current training step and $fade_{step}$ is the number of training steps for the 128 by 128 setting. When ${cur}_{s}step<{fade}_{s}step$, the loss function contains both low- and high-resolution losses. The low-resolution loss weights are then gradually reduced until only the high-resolution generated losses are relied upon. Training efficiency is significantly improved by this approach, with optimal performance at MS-COCO-14 256 × 256 achieved in just 8 hours on a 4 × RTX 3090.

## Experiments

### Datasets and metrics

**Fig 6:**
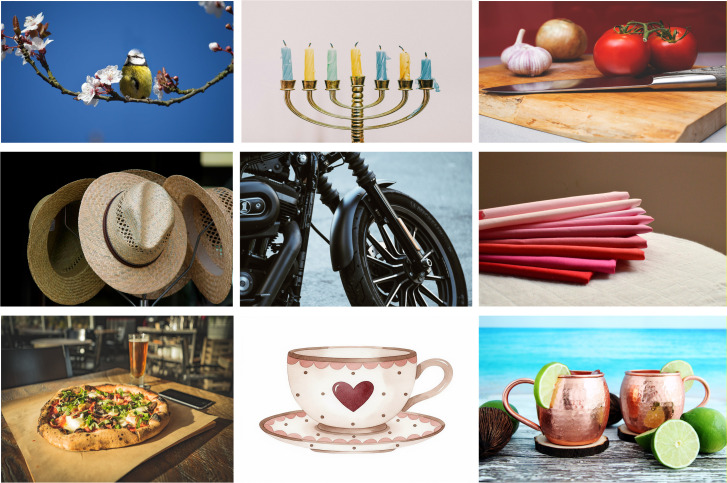
Partial examples on the AsHQ-10K dataset.

For the AI-generated advertisement task, we have built our own advertisement high-definition dataset (AsHQ-10K). Some of the images in its dataset are shown in Fig 6. The AdHQ-10K dataset contains a total of 10,000 ad images, about 60% of which come from manually selected high-resolution print ads and 40% of which come from key frames of more than 400 ad videos. All images were first screened for copyright compliance, then standardized to a resolution of 1024–2048 px, and JSON metadata such as source, industry category, major visual elements, and primary color were recorded for each image. Manual annotations were performed by trained annotators following unified guidelines. Each image was labeled with object category annotations, bounding box coordinates for foreground elements, and semantic background labels describing scene context. To ensure annotation quality, a two-stage verification process was employed in which annotations were independently reviewed and discrepancies were resolved through consensus. For model training, images were resized to 128 × 128 pixels during the initial training stage and progressively scaled to 256 × 256 pixels. Pixel values were normalized to the range [−1, 1]. Data augmentation techniques, including random horizontal flipping and mild color jittering, were applied to improve robustness and generalization. The data is divided into training, validation, and test sets by 8:1:1, which can be fine-tuned with the visual backbone network pre-trained on ImageNet and MS COCO for tasks such as advertisement scene categorization, character detection, product detection, logo detection, text detection, and text-to-graph generation. To prevent data leakage, splitting was performed at the advertisement identity level, ensuring that visually similar samples or multiple frames originating from the same advertisement did not appear across different subsets. This strategy guarantees fair evaluation and reliable performance assessment.

**Fig 7:**
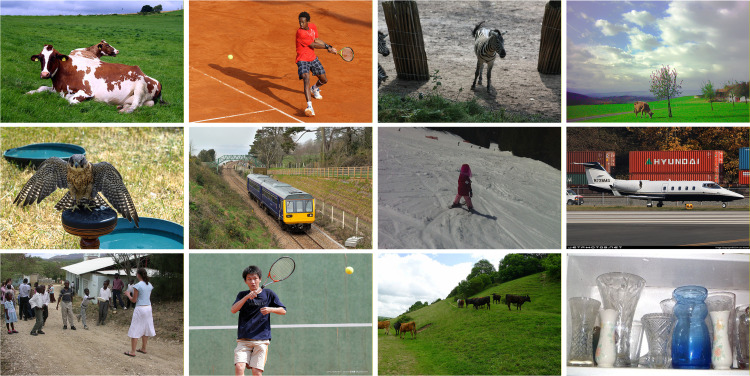
Partial examples on the COCO dataset.

In addition, in order to verify the generalization performance of the model. This paper also tests on the widely used MS-COCO dataset. Some of the images in its dataset are shown in Fig 7. This dataset contains 82,783 training images and 40,504 test images. Each image corresponds to 5 descriptive texts. The dataset contains 171 categories that demonstrate the diversity of objects in terms of scale, shape, and appearance, providing good data support for the model in generating high-quality and semantically rich images.

LAYOBJ-GAN is trained in two stages. In Stage 1 (text-to-layout), the Transformer-based layout generator is trained using teacher forcing with label smoothing (ε = 0.1). We use the Adam optimizer with a batch size of 64, an initial learning rate of 1e^−4^, β values of (0.9, 0.999), and train for approximately 50 epochs. In Stage 2 (layout-to-image), the generator is initialized from a pre-trained category-to-image model and trained adversarially together with image-level and region-level discriminators using Adam with batch size 32 and learning rates of 1e^−4^ for the generator and 4e^−4^ for the discriminators. The overall objective combines image-level adversarial loss, object-level adversarial loss, and text–image alignment loss, weighted by $\lambda_{img}=\text{1}.\text{0}$, $\lambda_{obj}=\text{1}.\text{0}$, and $\lambda_{align}=\text{0}.\text{5}$, respectively. To ensure statistical robustness, all experiments are repeated across three independent runs with different random seeds. We report mean and standard deviation for each metric and include statistical significance testing using paired t-tests with a significance threshold of p < 0.05.

In order to comprehensively assess the quality and text alignment of the images generated by the model, this paper employs three evaluation metrics, FID, R-precision, and SOA, to ensure that all aspects of the model’s performance are adequately measured.

This paper uses the widely known metric Fréchet Inception Distance (FID) to assess the quality of the generated images. The final layer of the pre-trained Inception-v3 network is used to obtain each image’s features, after which the feature distribution distance between the produced and real images is calculated. Better image quality is indicated by a lower FID.

In this study, we assess the text-image alignment using R-precision. This score is based on whether the generated image in the text candidate pool can be used to extract the input text. CLIP, a large-scale pre-trained image-text matching model, serves as the evaluator in this work. There will be one hundred candidate texts. If the input text appears as the first result returned, the generated image is deemed to be aligned with the input text.

To determine if the generated photos include the objects mentioned in the text, the Semantic Object Accuracy (SOA) metric is employed. It is composed of two sub-metrics: SOA-I measures the percentage of photos in which the target object is detected, and SOA-C measures the percentage of each detected object type. This measure includes an assessment of the model’s control over the position and quality of the images that are produced.

To ensure robust and statistically reliable evaluation, we adopt a stratified k-fold cross-validation protocol. For each evaluation metric, including FID, R-precision, SOA-I, and SOA-C, we report the mean and standard deviation across folds. In addition, 95% confidence intervals are computed to quantify performance variability and robustness. To assess the statistical significance of improvements over baseline methods, we conduct paired statistical tests between LAYOBJ-GAN and each competing model. When metric distributions satisfy normality assumptions, a paired t-test is applied; otherwise, the Wilcoxon signed-rank test is used. Improvements are considered statistically significant when p < 0.05.

### Ablation experiments

As shown in Table 1, the generated image with the help of background layout outperforms the object layout-only image in all metrics. The FID, R-precision, SOA-I, and SOA-C metrics of the generated images with only object layout are 7.53, 65.13, 55.27, and 35.03, respectively. After adding the background layout, the FID value decreased to 7.21, and the R-precision, SOA-I, and SOA-C metrics improved to 65.80, 55.91, and 35.62, respectively. This result shows that the background layout can provide additional contextual information for image generation. When the scenario structure is preplanned, the generator is more comprehensive in terms of layout and scene details, resulting in more realistic and text-consistent images. Background Layout facilitates the creation of image backgrounds. The advertisement’s background appears crisper and more detailed based on the background layout.

**Table 1:** Background generation and TL-Norm ablation experiments.

Object	Background	TL-norm	R-prec ↑	FID ↓	SOA-C ↑	SOA-I ↑
√			65.13	7.53	35.03	55.27
√	√		65.80	7.21	35.62	55.91
√	√	√	67.04	6.35	42.17	61.11

By contrasting TL-Norm with its baseline (the situation in Equation 5), its efficacy was confirmed. Table 1’s second and third rows demonstrate that every metric is better than the baseline. The addition of TL-Norm greatly enhances the model’s capacity to regulate object position and category when producing images, particularly on the SOA metrics. The SOA-I metric improves from 55.91 to 61.11, and the SOA-C metric improves from 35.62 to 42.17. These enhancements indicate that TL-Norm is more effective in achieving object knowledge migration from categories to images and enhances the generator’s performance in fine-grained control and object consistency.

This research generates images from the generated layouts using the second stage of the layout-to-image model in order to assess the Transformer-based layout generator’s performance. The transformer-based layout generator works better than the earlier LSTM-based approach, as Table 2 demonstrates. The FID increases to 9.35 from 29.71. In addition, this method shows competitiveness in comparison with real layouts (FID from 9.35 to 8.03 and R-precision from 67.24 to 70.87). The generated layouts even outperform the real layouts in terms of object quality metrics. This is due to the fact that the generator tends to produce larger bounding boxes than the real layouts, and therefore the corresponding objects are easier to generate in the final image.

**Table 2:** Advantages of using Transformer for modeling layout generation.

Model	R-prec ↑	FID ↓	SOA-C ↑	SOA-I ↑
Ground truth	70.87	8.03	48.25	66.39
LSTM	42.05	29.71	30.98	47.62
Transformer	67.24	9.35	49.11	68.35

**Fig 8:**
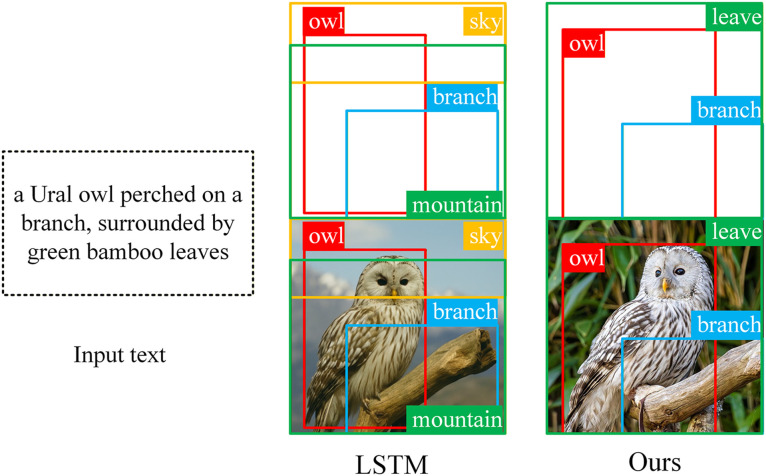
Advantages of using Transformer for modeling layout generation.

LSTM struggles to produce a thorough layout, as seen in Fig 8. The layout’s lower areas lack semantic categories, giving the finished image a blank background. This indicates its difficulty in capturing long-range dependencies, leading to duplication of similar targets. Semantic and spatial decoupling with white space at the bottom. Fig 8 may appear in public service announcements (PSAs) to advocate the protection of nature and other animals. However, the decoupling of the background and the owl can cause visual confusion for the advertiser and weaken the meaning of the advertisement. The boxes generated by Transformer are usually slightly larger than the real layout, which is in line with the design principle of “leaving enough safety zone and avoiding cropping key information” in advertisement creation. At the same time, the self-attention layer naturally produces multi-scale and multi-region interactions, allowing the final ad image to provide a more visually appealing hierarchy without sacrificing readability. When the ad script describes “a Ural owl perched on a branch, surrounded by bamboo leaves,” the Transformer puts the owl in the visual focus, the branch across the bottom diagonal, and the leaves/sky above. This layout matches the audience’s browsing behavior. All things considered, these findings demonstrate how well the Transformer-based layout generator performs when creating extensive layouts with a variety of spatial arrangements.

**Fig 9:**
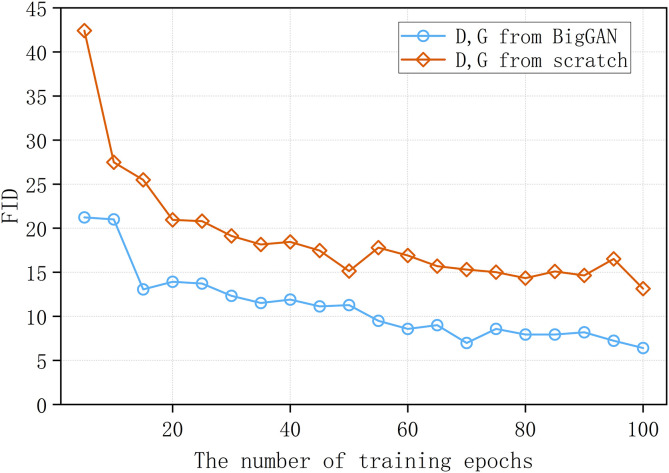
Impact on convergence speed after generating object models using pre-trained categories.

The generator and discriminator of BigGAN are used in this study as the source of object knowledge. When compared to the model that was trained from scratch, the outcomes of SOA are noticeably better, as indicated in Table 3. This demonstrates how object generation in T2I activities can benefit from category-to-image models as sources of object knowledge. It illustrates that utilizing a category-to-image model trained on a large-scale dataset can be used as effective a priori knowledge in layout-generated images to enhance the quality of object generation. And this study finds that model training can converge significantly faster based on this a priori model. As shown in Fig 9, when bootstrapping based on a pre-trained model prior, the effect of training the model for 10 epochs is equivalent to training it for 100 epochs from scratch, which is equivalent to a 10-fold speedup. And the model can converge to a lower FID. This acceleration effect is mainly due to the fact that the pre-trained models have learned rich object features on large-scale datasets. It enables LAYOBJ-GAN to effectively utilize this knowledge in the T2I task, resulting in a strong generative capability right from the initialization stage. Compared to training from scratch, the pre-trained model provides a higher starting point, allowing the model to learn layout and semantic features quickly and significantly reduce training time.

**Table 3:** Ablation experiments for object knowledge transfer.

D	G	R-prec ↑	FID ↓	SOA-C ↑	SOA-I ↑
		57.62	13.25	30.85	51.83
	√	64.00	8.41	36.25	55.78
√	√	67.04	6.35	42.17	61.11

### Comparison experiment

Table 4 displays the findings of the comparison between LAYOBJ-GAN and the state-of-the-art model on the MS-COCO dataset. It performs noticeably better than the second-best model, SSA-GAN, in terms of FID, going from 15.85 to 9.3. LAYOBJ-GAN performs better than the second-best model, DM-GAN, in terms of R-precision, going from 65.4% to 67.2%. This suggests that the generated images are more semantically consistent with the input text. In terms of SOA-I/C measures, LAYOBJ-GAN also performs better than DAE-GAN, the next best model, rising from 53.4/34.1 to 68.35/49.05. LAYOBJ-GAN is able to generate objects mentioned in text better in images. While ControlNet benefits from a powerful diffusion backbone and achieves strong visual fidelity, it relies on externally provided control maps and lacks an explicit text-to-layout generation mechanism. In contrast, LAYOBJ-GAN predicts background-aware layouts directly from text and provides finer object-level controllability without requiring manual or auxiliary conditioning inputs. This highlights a trade-off between diffusion-based image quality and layout-predictive controllability and shows that LAYOBJ-GAN is also able to generate images with consistent text and layout on the MS-COCO dataset with significantly improved image quality.

**Table 4:** Comparison of quantitative results of LAYOBJ-GAN with other correlation methods on MS COCO dataset.

Method	R-prec ↑	FID ↓	SOA-C ↑	SOA-I ↑
Real Images	83.1	2.3	75.6	83.05
ControlNet	41.5	28.11	20.2	44.2
AttnGAN	52	30.6	25.8	43.25
DM-GAN	65.4	24.2	33.8	53.3
Obj-GAN	58.4	26.15	31	49.85
R-GAN	–	24.7	–	–
DAE-GAN	64.6	24.95	34.1	53.4
DF-GAN	38.25	22.1	20.8	40.75
SSA-GAN	57.9	15.85	29.3	50.7
**LAYOBJ-GAN**	**67.2**	**9.3**	**49.05**	**68.35**

To further assess the performance, this paper further conducts a manual evaluation to compare the actual generation results of LAYOBJ-GAN with other state-of-the-art models on the self-constructed AsHQ-10K dataset. One hundred descriptions and their corresponding images generated by Obj-GAN, SSA-GAN, and LAYOBJ-GAN were randomly selected, and then the users were asked to select the best (top-1) image among the candidates based on the image quality and the consistency between the text and the image. Table 5 shows that LAYOBJ-GAN obtains the highest selection of 77.1%; in contrast, Obj-GAN and SSA-GAN have much lower selection rates than LAYOBJ-GAN, indicating that LAYOBJ-GAN performs better in subjective user evaluation.

**Table 5:** Comparison results of LAYOBJ-GAN with other methods on manual evaluation metrics.

	Obj-GAN	SSA-GAN	LAYOBJ-GAN
top-1	4.6%	18.6%	77.1%

**Fig 10:**
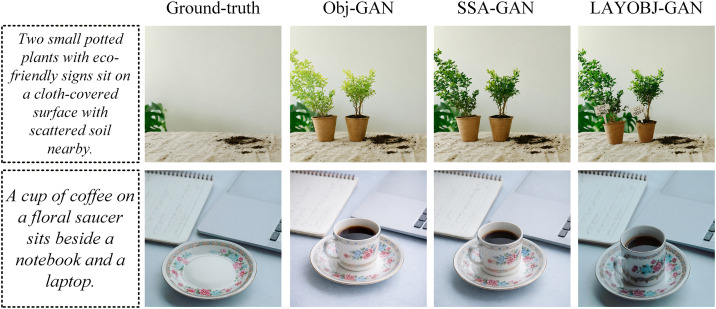
Comparison of qualitative results between LAYOBJ-GAN and other methods.

The images produced by the traditional two-stage model Obj-GAN and the cutting-edge single-stage model SSA-GAN are displayed in Fig 10. In the “two small potted plants with eco-friendly signs” scenario, the image generated by Obj-GAN exhibits a yellowish color cast, with plant details appearing slightly blurred and failing to fully restore the lighting and shadow effects present in the real image. While SSA-GAN improved brightness, it still appeared flat, lacking detail and depth. In contrast, LAYOBJ-GAN successfully restored the plants’ true colors and intricate details, showcasing more natural light-shadow transitions and distinct contours, making the entire scene feel closer to the real world. In the scene depicting “A cup of coffee on a floral saucer sits beside a notebook and a laptop,” Obj-GAN’s generated image appears overly simplistic and lacks detail. The intricate features of the coffee cup and saucer are blurred, failing to perfectly render the objects’ textures. SSA-GAN’s image shows slight improvement but still lacks necessary refinement, with overly monotonous colors and insufficient depth. LAYOBJ-GAN delivers the best results in this scenario, successfully reproducing the textures of the coffee cup, saucer, notebook, and laptop. It features rich, layered colors and sharper details. In summary, LAYOBJ-GAN outperforms both Obj-GAN and SSA-GAN in both scenarios. LAYOBJ-GAN delivers more natural and realistic results in detail restoration, color reproduction, and lighting effects. This grants it a significant advantage in generating high-quality images, particularly demonstrating greater potential in commercial advertising and creative content generation. In contrast, Obj-GAN and SSA-GAN exhibit substantial gaps in detail rendering and color gradation, failing to achieve satisfactory outcomes.

**Fig 11:**
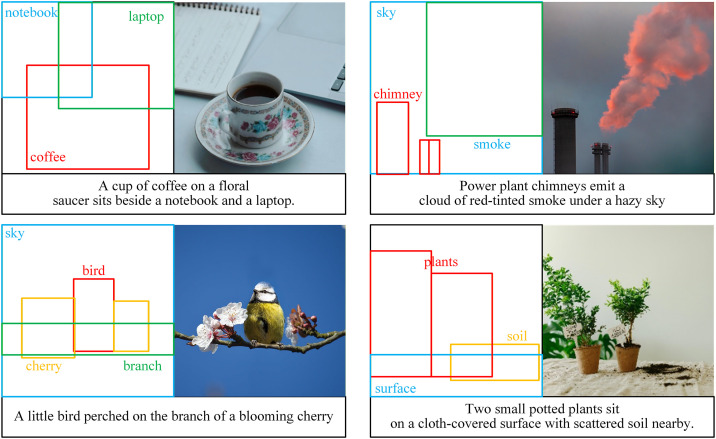
The intermediate background, object layout, and matching final image were produced by LAYOBJ-GAN.

The intermediate backdrop arrangement and accompanying images produced by LAYOBJ-GAN are shown in Fig 11. It can be observed that LAYOBJ-GAN is able to generate a rougher background layout by comprehensively planning the contents and their relative positions in the scene based on the input text description. This layout structure provides the initial spatial distribution and object composition for the generated images. This allows the model to have a higher degree of control during the generation process, and in a second stage, the control of the layout is realized as well as the generation of better-quality images.

It is also important to consider broader issues related to data bias, generalization, and external validation. Potential bias may arise from the underlying training data, such as over-representation of certain product types, visual styles, or cultural aesthetics. These biases may influence generated content and limit diversity in output. While our method does not perform decision-making or personalization, careful dataset curation and diversity-aware sampling are necessary to mitigate biased visual representations in downstream applications. External validation is essential to assess robustness beyond the datasets used in this study. Although our experiments include both a proprietary advertisement dataset and the public MS-COCO benchmark, future work should evaluate performance on advertisements from different industries, regions, and visual styles. The proposed framework is not restricted to advertisement imagery and can be generalized to other layout-sensitive generation tasks, such as product visualization, e-commerce content generation, and scene illustration. With appropriate retraining, the system can also be adapted to different image domains and resolutions.

## Conclusion

For the task of AI-generated advertisement, this paper proposes a Background and Object GAN (LAYOBJ-GAN) for layout-controllable text-to-generate images, aiming to address the limitations of existing methods in generating images of complex scenes. The following fundamental steps make up the suggested two-phase approach: The layout-to-image phase uses the pre-trained knowledge of the single-object image generation model for object knowledge transfer to improve the quality of object generation. Initially, layout generation is redesigned in the text-to-layout phase to combine the background layout and object layout to generate a complete image structure. Technically, this work first designs a Transformer sequence-to-sequence-based layout generator to model complex layout distributions. Then a fine-grained text-layout interaction module is proposed to realize the efficient migration of material knowledge. Numerous quantitative and qualitative tests confirm LAYOBJ-GAN’s outstanding capacity to produce realistic and complicated scene images, particularly in terms of image quality and position controllability. Although this work is motivated by advertisement image generation, the proposed framework is not restricted to this domain. The layout-aware generation paradigm can be directly applied to other tasks requiring structured spatial control, such as e-commerce visualization, scene synthesis, and controllable content generation.

Despite its effectiveness, the proposed framework has several limitations. First, layout quality degrades as the number of objects increases; in complex multi-object scenes, the layout generator may suffer from positional deviations or missing elements, reducing consistency with the input description. Second, the overall quality of generated images remains constrained by the limitations of GAN-based generation and the scale of available pre-training data, leaving room for improvement in fine-grained details and realism. Third, the autoregressive layout generation process is susceptible to error accumulation due to teacher-forcing during training, leading to discrepancies between training and inference and potential degradation in downstream image quality. As a GAN-based method, image quality is also constrained by pre-training scale and may exhibit artifacts in challenging visual conditions. In terms of computational cost, the two-stage design increases training overhead and requires multi-GPU resources, although inference remains efficient and suitable for offline or cloud-based deployment.

Future work will focus on improving robustness and scalability in complex scenes by incorporating more diverse training data and increasing layout model capacity to better capture object interactions. In addition, integrating large-scale pre-trained text-to-image foundation models as generative priors offers a promising direction to further enhance image fidelity and semantic consistency in both layout and image generation stages. Integrating diffusion-based generative models can further enhance image fidelity and competitiveness.
